# Under five mortality patterns and associated maternal risk factors in sub-Saharan Africa: A multi-country analysis

**DOI:** 10.1371/journal.pone.0205977

**Published:** 2018-10-25

**Authors:** Sanni Yaya, Ghose Bishwajit, Friday Okonofua, Olalekan A. Uthman

**Affiliations:** 1 School of International Development and Global Studies, University of Ottawa, Ottawa, Canada; 2 Women’s Health and Action Research Centre, Benin City, Nigeria; 3 Warwick Centre for Applied Health Research and Delivery (WCAHRD), Division of Health Sciences, Warwick Medical School, University of Warwick, Coventry, United Kingdom; TNO, NETHERLANDS

## Abstract

**Background:**

Under-5 mortality rate in the sub-Saharan region has remained unabated. Worse still, information on the regional trend and associated determinants are not readily available. Knowledge of the trend and determinants of under-5 mortality are essential for effective design of intervention programmes that will enhance their survival. We aimed to examine the mortality patterns in under-5 children and maternal factors associated with under-5 deaths.

**Methods:**

Demographic and Health Survey (DHS) data from five sub-Sahara Africa countries; Chad, Democratic Republic of Congo, Mali, Niger and Zimbabwe were used in this study. The sample size consisted of 68,085 women aged 15–49 years with at least one history of childbirth. The outcome variable was under-five mortality rate. Relevant information on maternal factors were extracted for analysis. Multivariable Cox proportional hazards regression was used to model maternal factors associated with under-five mortality.

**Results:**

The current under-5 mortality rate (per 1,000 live births) was; 133 in Republic of Chad, 104 in Democratic Republic of Congo, 95 in Mali, 127 in Niger, and 69 in Zimbabwe. Several maternal and child level factors were found to be significantly associated with under-five mortality. Lack of spousal support (not currently married) resulted to increase in under-five mortality (Chad- Hazard Ratio [HR] = 1.11, 95%CI = 0.97–1.25; DR Congo- HR = 1.24, 95%CI = 1.11–1.40; Mali- HR = 2.43, 95%CI = 1.63–3.64; Niger- HR = 1.59, 95%CI = 1.24–2.03; Zimbabwe- HR = 1.33, 95%CI = 1.06–1.67). Delivery by caesarean section was significantly associated with under-five mortality (Chad- HR = 1.32, 95%CI = 1.00–1.77; DR Congo- HR = 1.20, 95%CI = 1.01–1.43; Mali- HR = 1.42, 95%CI = 1.08–1.85; Niger- HR = 1.43, 95%CI = 1.06–1.92; Zimbabwe- HR = 1.49, 95%CI = 1.03–2.15).

**Conclusion:**

Despite concerted effort by government and several stakeholders in health to improve childhood survival, the rate of under-5 mortality is still high. Our findings provided evidence on the contribution of maternal age, place of residence, household wealth index, level of education, employment, marital status, religious background, birth type, birth order and interval, sex and size of child, place and mode of delivery, to Under-5 mortality rate in SSA. The position of prominent risk factors for under-five mortality should be addressed through effective design of timely and efficient intervention aimed at reducing childhood mortality.

## Background

Mortality of children under-5 years is a critical issue to demographers and public health experts and a core indicator of the development of families, societies and the world at large. At policymaking level, it reflects the macroeconomic and public health priorities and values of a country. Global under-5 mortality rates have declined by half from 91 deaths per 1,000 live births in 1990 to 43 deaths per 1,000 live births in 2015 [1]. The World Health Organization (WHO) has estimated an average yearly rate of decline for under-5 mortality from about 2% per annum from 1990–2000 to approximately 4% as of 2000–2015 [1]. However, the improvement was not enough to meet the Millennium Development Goal 4 (MDG-4) targeted at reducing child mortality.

The Sub-Sahara region has age-long prevalence of under-5 mortality, despite increasing health interventions in the region [2]. Classifying countries by hierarchical mortality strata, the risk of under-5 mortality was reportedly highest in the seven sub-Saharan African countries (Nigeria, Somalia, Angola, Central African Republic, Mali Sierra Leone and Chad) out of 194 countries. Jointly, they accounted for about one-fifth (1·1 million out of 5·9) million) of under-5 mortality worldwide [2]. Though the current statistics are worrisome and the 2015 MDG-4 target of two-thirds reduction were far from being achieved, it suffices to note that child survival has improved significantly compared to past decades [3]. The success is also a product of the creation and implementation of the MDGs, subsequent scaling up of numerous life-saving interventions and the resultant increase in developmental support [3,4].

The beginning of 2016 saw a reinforcement of the fight against issue of under-5 mortality by the introduction of Sustainable Development Goals (SDGs) [5]. The implementation of SDGs targets an under-5 mortality rate of 25 per 1000 live births in all countries by 2030 [5]. However, this target is not insurmountable for sub-Saharan Africa (SSA) countries, though high under-5 mortality rate still persist in contrast with other developing regions that have made remarkable success in this regard [6]. There are several methods to address this problem such as; immunization, prevention, early treatment and control of malaria and diarrhea as well as other prevalent childhood diseases. These could have impact in the reduction of under-5 mortality. With large disparities in the trend of child mortality across many factors and high incidence of child mortality in SSA countries, the demand for greater effort to improve infant survival is paramount. The risk of neonatal, infant and child’s death has been known to have intimate link with maternal attributes [7]. Several evidence-based studies have investigated the determinants of neonatal, child and under-5 mortalities as to proffer long-term solutions.

The use of a single country data or singleton births when conducting childhood mortality research has limited the generalizability of many reports in some dimension. For example, Abir *et al*., [8], Ezeh *et al*., [9], Ezeh [10], Akinyemi *et al*., [11], Zhu *et al*., [12] amongst others conducted studies on determinants of neonatal and under-5 mortality using only singleton data, leaving out multiple births. Under-5 mortality rate is generally known to represent the probability of a child who survives to age one, dying between age one and age five [13]. Such estimates should include both singleton and multiple births for comprehensive comparisons. The sum of statistics revealed approximately half of the global child mortality occurring in Africa and about 25,000 of under-5 daily deaths are concentrated in SSA region and South Asia [13]. Elimination of avoidable child mortality requires information about existing distribution of prominent causes of deaths. To date, there remains discrepancies related to the series of reports provided from several large data samples contribute to the problems of determining true estimates on under-five death rates in SSA region. Therefore, in this study, we explored maternal characteristics associated with under-5 mortality at regional level.

## Methods

### Data sources

The individual woman component of Demographic Health Survey (DHS) data from five sub-Sahara Africa countries including Chad, Democratic Republic of Congo, Mali, Niger and Zimbabwe were analyzed in this study. The data was retrieved after receiving approval form the authorized body. More so, the child variables form the data was extracted to investigate the trends and factors of under-5 death rate in sub-Sahara region in general. The DHS 2015 of 17,719 respondents was utilized for Republic of Chad, while DHS 2014 data of 18,827 respondents was used for Democratic Republic of Congo. In addition, DHS 2013 made up of 10,424 respondents was utilized for Mali, and a sample of 11,160 respondents from DHS 2012 for Niger and finally Zimbabwe DHS 2015 of 9,955 respondents was also used. Interestingly, DHS data were based on nationally representative sample of women of reproductive age (15–49 years) selected using a stratified two-stage cluster sampling technique. Key reproductive health variables were collected in the survey by trained field workers through structured interviewers’ administered instruments. Maternity history was also elicited from the women’s questionnaire, with data from the birth history recoded into separate records for individual children reported by the respondents.

### Selection criteria for study countries

These countries were selected based on previous reports of under-5 mortality rate by United Nations Children Fund/World Health Organization. The study showed that the selected countries (Chad, DR Congo, Mali, Niger and Zimbabwe) had leading mortality rate in the sub-regional strata. In addition, considering geographical diversity, at least one country was selected from each stratum (West, Central and Southern) of sub-Sahara Africa [14].

### Determination of under-5 mortality

The computation of under-5 mortality was based on data extracted from the birth history section of the individual women questionnaire. For each live birth to a woman, some questions on year and month of birth, whether the child is dead or alive, the age at death (if the child was dead), sex of the child. The under-5 mortality rate was estimated from the data in the birth history from a child’s birth, survivorship status, and age at death (if dead). The under-5 mortality rates were calculated using an approach similar to Rutstein and Rojas [15]. The data used for estimating under-5 mortality rates in this study was based on live births reported by the respondents. The outcome variable for this paper was the risk of under-5 death which was measured from the duration of survival in months. This was defined as the risk of a child dying before reaching its fifth birthday. The analysis was based on children’s data, particularly, all deaths from age 0 to 59 months.

### Study variables

The maternal level variables in this study included age in years (15–19,20–24…45–49), place of residence (urban vs rural), geographical region, religion (Christianity, Islam and other religious beliefs), level of education (no formal education, primary, secondary and higher), wealth index (poorest, poorer, middle, richer, richest), age at first birth, marital status (currently married/in union vs not currently married), employment status (currently working or not). The child characteristics were birth type (singleton or multiple), sex of the child (male or female), place of delivery (home vs health facility), mode of delivery (caesarean section or not caesarean section), size of the child (large, average, small), preceding birth interval (<18, 18–24 and >24 months) and birth order (1–4 vs 5 and above). The model by Moseley [16] on systematic conceptual framework was used in the selection of explanatory factors in this study. Here, our study utilized this conceptual framework as the basis for identifying vital risk factors for under-five mortality in sub-Sahara region.

### Ethical considerations

We did the analyses using publicly available data from demographic health surveys. Ethical procedures were the responsibility of the institutions that commissioned, funded, or managed the surveys. All DHS surveys are approved by ICF international as well as an Institutional Review Board (IRB) in respective country to ensure that the protocols are following the U.S. Department of Health and Human Services regulations for the protection of human subjects.

### Data analysis

Descriptive summary statistics and percentages were used to present distribution of maternal and child characteristics. Under-5 mortality was computed as the probability of a child dying before 5 completed years. To examine the effects of the explanatory variables on under-5 mortality, univariate Cox proportional hazards regression models were fitted separately for each country DHS data sets. Based on the variables that were statistically significant, a set of multivariable regression models were computed separately for each country. Cox regression is used to analyze time-to-event data, that is, the response is the time an individual takes to present the outcome of interest. In this study, children that were still alive at age 5 years are assigned the total length of time of the follow-up, and are treated as *censored*, meaning that until the time of the end of the follow-up they are alive.

The level of statistical significance was set at 5%. All data analyses were conducted using STATA 14.0 (Statacorp, College Station, Texas, United States of America).

We used geographical factor detector model, spatial stratified heterogeneity q-statistic [17] (Power of Determinant) to assess the impact of social economic factors (i.e. illiteracy rate, unemployment rate, and poverty rate) on the spatial pattern of under-five mortality rate using R ‘geodetector’. F-test was used to compare whether the accumulated variance of each strata is significantly different from the variance of the entire country.

## Results

### Sample characteristics

The distribution of respondents’ characteristics was presented in Table 1. The mean age of respondents was approximately 28.5 and age at first birth was about 18years, with women in the 40–44 and 45–49 age groups having the least number of respondents. For age groups, women aged 40–44 and 45–49 years were least throughout the study. The distribution of age interval showed that younger women had more representation. The distribution of the place of residence showed that SSA countries had more respondents from rural areas at least 55%. There were variations in educational attainment across the countries of study as about two-third of respondents from Chad had no formal education, Mali and Niger accounted for about three-quarters of women with no formal education. Interestingly, in Zimbabwe only 1.1% had no formal education, while Democratic Republic of Congo reported 17.8% women having no formal education. For women with a minimum of secondary education, Chad was least while Zimbabwe outscored other countries having most educated respondents. For religious beliefs, about 64.7% of Chadian respondents and 93.2% of Malians belonged to the Islamic sect respectively. But Christianity is predominantly reported for Democratic Republic of Congo (92.7%) and Zimbabwe (94.3%). This showed broad disparities in religious background in the study countries. From the results on wealth index, there were wide differences between low and high economic class among the study population. In each country, a minimum of 30% of the respondents was reported below middle class. Furthermore, the currently working section of the respondents varied widely, as Democratic Republic of Congo reported more than two-third of the respondents currently employed. However, this was as low as 27.1% in Niger, 39.1 in Chad and approximately 40% in both Mali and Zimbabwe respectively.

**Table 1:** Background characteristics of study respondents (n = 68,085).

Variable	Chad-DHS 2015 (n = 17,719)	DR Congo-DHS 2014 (n = 18,827)	Mali-DHS 2013 (n = 10,424)	Niger-DHS 2012 (n = 11,160)	Zimbabwe-2015 (n = 9,955)
**Maternal characteristics**					
Age at first birth	17.9±3.6	19.2±3.8	18.8±4.2	18.3±3.6	19.7±3.3
**Age(years)**					
15–19	3889 (21.9)	3981 (21.1)	1918 (18.4)	1901 (17.0)	2156 (21.7)
20–24	2995 (16.9)	3680 (19.5)	1880 (18.0)	1968 (17.6)	1782 (17.9)
25–29	3287 (18.6)	3485 (18.5)	2075 (19.9)	2281 (20.4)	1656 (16.6)
30–34	2540 (14.3)	2572 (13.7)	1657 (15.9)	1845 (16.5)	1591 (16.0)
35–39	2115 (11.9)	2191 (11.6)	1330 (12.8)	1421 (12.7)	1209 (12.1)
40–44	1545 (8.7)	1595 (8.5)	902 (8.7)	995 (8.9)	966 (9.7)
45–49	1348 (7.6)	1323 (7.0)	662 (6.4)	749 (6.7)	595 (6.0)
**Mean (±SD) age**	28.6±9.3	28.3±9.4	28.5±8.9	28.7±8.9	28.5±9.3
**Place of residence**					
Urban	4285 (24.2)	6827 (36.3)	3262 (31.3)	3400 (30.5)	4521 (45.4)
Rural	13434 (75.8)	12000 (63.7)	7162 (68.7)	7760 (69.5)	5434 (54.6)
**Level of education**					
No formal	12195 (68.8)	3357 (17.8)	7721 (74.1)	8326 (74.8)	106 (1.1)
Primary	3311 (18.7)	7320 (38.9)	1014 (9.7)	1439 (12.9)	2385 (24.0)
Secondary	2080 (11.7)	7589 (40.3)	1537 (14.7)	1256 (11.3)	6637 (66.7)
Higher	133 (0.8)	561 (3.0)	152 (1.5)	117 (1.1)	827 (8.3)
**Religion**					
Christianity	5663 (32.0)	17459 (92.7)	408 (3.9)	No data	9385 (94.3)
Islam	11459 (64.7)	290 (1.5)	9715 (93.2)	No data	30 (0.3)
Others	597 (3.4)	1078 (5.7)	301 (2.9)	No data	540 (5.4)
**Wealth index**					
Poorest	3183 (18.0)	4366 (23.2)	1828 (17.5)	1743 (15.6)	1499 (15.1)
Poorer	3247 (18.3)	3740 (19.9)	1872 (18.0)	1759 (15.8)	1452 (14.6)
Middle	3591 (20.3)	3655 (19.4)	1855 (17.8)	1910 (17.1)	1549 (15.6)
Richer	4040 (22.8)	3390 (18.0)	1977 (19.0)	2093 (18.8)	2558 (25.7)
Richest	3658 (20.6)	3676 (19.5)	2892 (27.7)	3655 (32.8)	2897 (29.1)
**Currently working**					
Yes	6831 (39.1)	12830 (68.4)	4436 (42.6)	3014 (27.1)	4104 (41.2)
No	10644 (60.9)	5932 (31.6)	5988 (57.4)	8121 (72.9)	5851 (58.8)
**Marital status**					
Currently married/in union	13439 (75.8)	12448 (66.1)	8737 (83.8)	9509 (85.2)	6015 (60.4)
Not currently married	4280 (24.2)	6379 (33.9)	1687 (16.2)	1651 (14.8)	3940 (39.6)
**Child characteristic**					
**Birth type**					
Singleton	13936 (78.7)	13884 (73.7)	8323 (79.8)	9028 (80.9)	7111 (71.4)
Multiple	3783 (21.3)	4943 (26.3)	2101 (20.2)	2132 (19.1)	2844 (28.6)
**Sex of child**					
Male	7144 (50.5)	7106 (50.1)	4381 (51.7)	4602 (50.0)	3701 (51.0)
Female	7012 (49.5)	7076 (49.9)	4099 (48.3)	4607 (50.0)	3552 (49.0)
**Place of delivery**					
Home	8776 (49.5)	2676 (14.2)	2663 (25.5)	4582 (41.1)	734 (7.4)
Health facility	8943 (50.5)	16151 (85.8)	7761 (74.5)	6578 (58.9)	9221 (92.6)
**Mode of delivery**					
Caesarean section	146 (1.3)	558 (4.9)	235 (3.5)	159 (2.1)	328 (6.8)
Non-caesarean section	10945 (98.7)	10720 (95.1)	6488 (96.5)	7440 (97.9)	4502 (93.2)
**Size of child**					
Large	4856 (44.1)	5517 (49.7)	2812 (43.7)	1684 (22.8)	1765 (36.6)
Average	3057 (27.8)	4286 (38.6)	2729 (42.4)	3929 (53.2)	2357 (48.9)
Small	3097 (28.1)	1296 (11.7)	897 (13.9)	1776 (24.0)	696 (14.4)
**Birth interval (months)**					
<18	1261 (10.2)	990 (8.5)	495 (7.0)	541 (6.7)	195 (3.5)
18–24	2430 (19.6)	2073 (17.9)	1017 (14.4)	1420 (17.6)	427 (7.7)
>24	8722 (70.3)	8533 (73.6)	5570 (78.7)	6088 (75.6)	4942 (88.8)
**Birth order**					
1–4	6987 (49.4)	8549 (45.2)	5330 (51.1)	4721 (42.3)	6171 (62.0)
5 and more	7170 (50.6)	10278 (54.8)	5094 (48.9)	6439 (57.7)	3784 (38.0)

**Fig 1:**
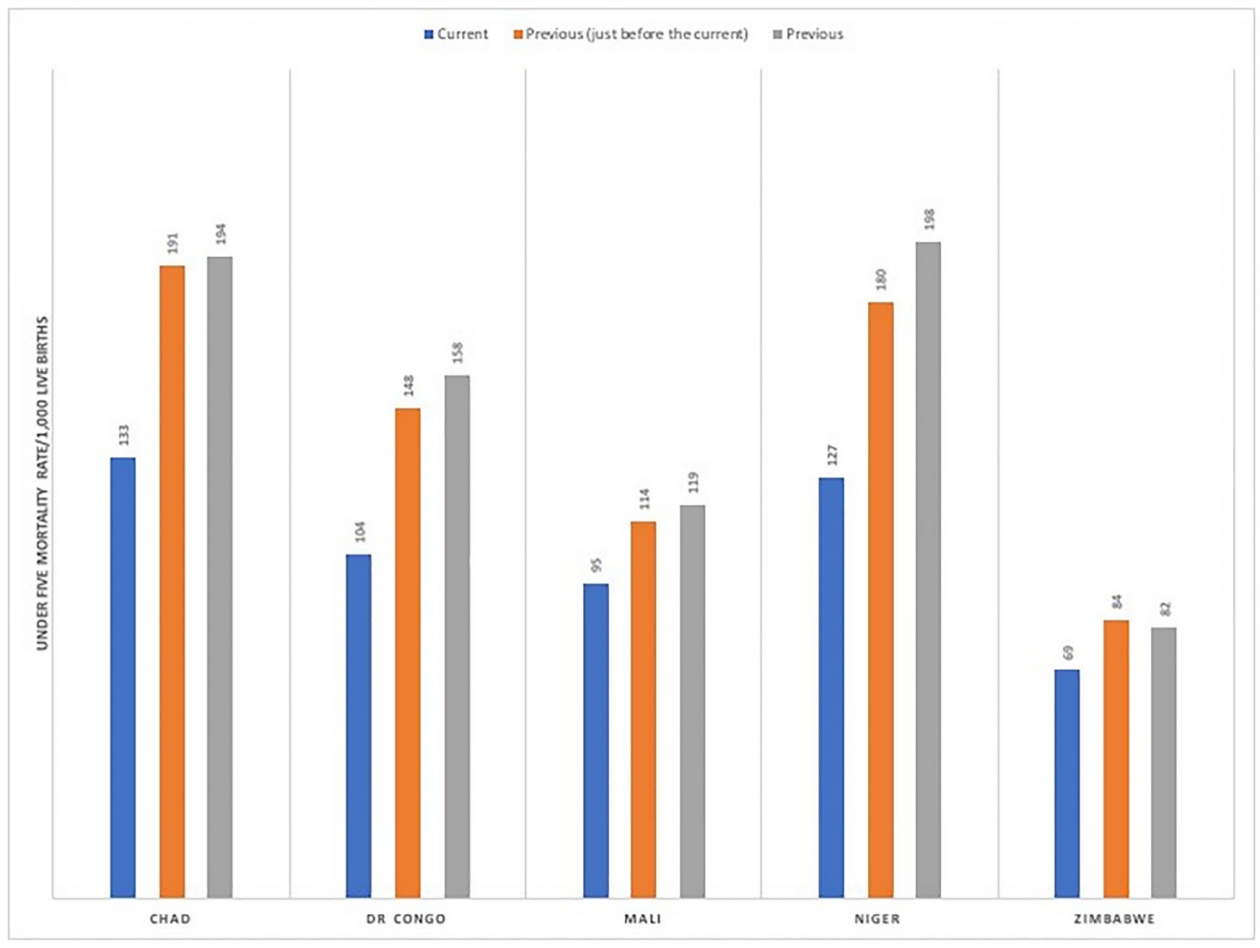
Under-five mortality rate, by countries.

From Fig 1 the trend in under-five mortality was presented across various countries in sub-Sahara region. The current under-five mortality rate was highest in Chad (133 per 1000 live births), followed by Niger (127 per 1000 live births); and lowest in Zimbabwe (69 per 1000 live births). However, the patterns showed a reduction in the trend of under-five mortality from previous years.

Marital status of women showed that up to 85.2% were currently married or in union. Among the countries studied, the least proportion of women currently married or in union was above 60%. More so, from the under-five characteristics, above 71% of birth type was singleton which indicated high survival chances of under-five children. The sex of under-five children had little or no difference in all the countries under study. The places of delivery varied widely across the countries. In Zimbabwe, about 93% of the respondents reported to have delivered in health facilities, while in Chad it was reported as 50.5%, Democratic Republic of Congo accounted for 85.8%, Mali 74.5% and Niger was 58.9%. Delivery by caesarean section was reported lowest in Chad (1.3%), while Zimbabwe had the highest proportion of 6.8%. More than two-third of the children was above average size. While Democratic Republic of Congo reported as low as 11.7% of small size children. In addition to the children characteristics, not greater than 10% had birth interval less than 18 months and first to fourth birth order was about 50% throughout the countries under study.

### Factors associated with under five mortality

The results of multivariable Cox hazard regression to investigate the determinants of under-five mortality are presented for each country (see Table 2). There were differentials in under-five mortality for every unit increase in the age at first birth. In Chad, there was reduction in under-five mortality for every unit increase in the age at first birth. On the contrary, an increase in age at first birth was associated with under-five death. Furthermore, maternal age was significantly associated with under-five mortality in sub-Sahara Africa countries. Women at higher age groups had increase in under-five mortality for all the countries under study, compared to women aged 15–19 years and this was statistically significant. More so, the place of residence revealed that the under-five mortality was significantly higher in rural areas compare to the urban counterpart (Chad- HR = 1.11, 95%CI = 1.01–1.19; DR Congo- HR = 1.29, 95%CI = 1.02–1.57; Mali- HR = 1.28, 95%CI = 1.01–1.64; Niger- HR = 1.14, 95%CI = 1.01–1.33; Zimbabwe- HR = 1.01, 95%CI = 0.83–1.20). Multivariable Cox hazard regression showed that level of level of education and religious beliefs were significantly associated with under-five mortality. Increase in the level educational attainment reduced under-five mortality when compared to women with no formal education, after controlling for other confounder variables. The religious beliefs did not show any significant association with under-five mortality after controlling for other covariates. In addition, wealth index was significantly associated with under-five mortality.

**Table 2:** Multivariable Cox proportional regression model of the factors associated with under-five mortality sub-Sahara Africa countries.

Variable	Chad	DR Congo	Mali	Niger	Zimbabwe
	HR (95%CI)	HR (95%CI)	HR (95%CI)	HR (95%CI)	HR (95%CI)
**Maternal characteristics**					
Age at first birth	0.89 (0.68–1.23)	ns	1.07 (1.05–1.09)*	ns	1.07 (1.03–1.09)*
**Age(years)**					
15–19	1.00	1.00	1.00	1.00	1.00
20–24	0.61 (0.81–1.56)	0.61 (0.44–1.16)	0.96 (0.53–2.64)	0.42 (0.27–1.64)	0.71 (0.60–1.39)
25–29	1.03 (0.91–1.47)	0.94 (0.32–2.62)	1.12 (0.99–1.36)	1.25 (0.56–1.88)	1.21 (0.81–1.85)
30–34	0.83 (0.76–1.42)	1.38 (1.27–1.74)*	1.37 (1.12–1.94)*	1.47 (1.11–1.96)*	0.83 (0.58–1.64)
35–39	0.86 (0.62–1.34)	0.93 (0.73–1.46)	1.28 (1.09–2.17)*	1.16 (0.70–2.24)	0.97 (0.66–1.49)
40–44	1.25 (1.19–1.32)*	1.28 (1.20–1.39)*	1.11 (0.80–1.16)	2.15 (1.10–3.24)*	1.23 (1.05–1.38)*
45–49	1.21 (1.16–1.38)*	1.25 (1.17–1.36)*	1.08 (0.85–1.53)	2.04 (1.09–3.21)*	1.30 (1.03–1.83)*
**Place of residence**					
Urban	1.00	1.00	1.00	1.00	1.00
Rural	1.11 (1.01–1.19)*	1.29 (1.02–1.57)*	1.28 (1.01–1.64)*	1.14 (1.01–1.33)*	1.01 (0.83–1.20)
**Level of education**					
No formal	1.00	ns	ns	ns	1.00
Primary	0.93 (0.85–1.01)	ns	ns	ns	0.61 (0.38–1.00)
Secondary	0.99 (0.85–1.15)	ns	ns	ns	0.62 (0.38–0.99)*
Higher	0.41 (0.15–1.12)	ns	ns	ns	0.47 (0.23–0.96)*
**Religion**					
Christianity	1.00	ns	ns	ns	1.00
Islam	0.96 (0.84–1.10)	ns	ns	ns	2.41 (0.33–17.25)
Others	0.95 (0.79–1.13)	ns	ns	ns	1.01 (0.75–1.35)
**Wealth index**					
Poorest	1.00	1.00	1.00	1.00	ns
Poorer	0.99 (0.89–1.09)	0.96 (0.87–1.07)	0.99 (0.85–1.14)	0.93 (0.83–1.05)	ns
Middle	0.97 (0.88–1.07)	0.92 (0.83–1.01)	1.00 (0.86–1.15)	0.97 (0.86–1.08)	ns
Richer	0.94 (0.85–1.04)	0.89 (0.79–0.99)*	0.94 (0.79–1.11)	1.01 (0.89–1.14)	ns
Richest	0.99 (0.86–1.15)	0.78 (0.65–0.94)*	0.88 (0.70–1.11)	0.84 (0.61–0.95)*	ns
**Currently working**					
Yes	ns	ns	0.98 (0.89–1.08)	0.96 (0.88–1.04)	1.02 (0.87–1.18)
No	ns	ns	1.00	1.00	1.00
**Marital status**					
Currently married/in union	1.00	1.00	1.00	1.00	1.00
Not currently married	1.11 (0.97–1.25)	1.24 (1.11–1.40)*	2.43 (1.63–3.64)*	1.59 (1.24–2.03)*	1.33 (1.06–1.67)*
**Child characteristics**					
**Birth type**					
Singleton	ns	ns	ns	1.00	1.00
Multiple	ns	ns	ns	1.14 (1.04–1.31)*	1.19 (1.01–1.57)*
**Sex of child**					
Male	1.00	1.00	ns	ns	1.00
Female	0.96 (0.91–1.02)	0.99 (0.93–1.07)	ns	ns	1.05 (0.91–1.22)
**Place of delivery**					
Home	1.00	ns	1.00	ns	ns
Health facility	0.99 (0.90–1.08)	ns	0.99 (0.89–1.11)	ns	ns
**Mode of delivery**					
Caesarean section	1.32 (1.00–1.77)*	1.20 (1.01–1.43)*	1.42 (1.08–1.85)*	1.43 (1.06–1.92)*	1.49 (1.03–2.15)*
Non-caesarean section	1.00	1.00	1.00	1.00	1.00
**Size of child**					
Large	1.00	1.00	1.00	1.00	ns
Average	1.04 (0.96–1.13)	1.02 (0.95–1.10)	0.96 (0.86–1.06)	1.03 (0.93–1.14)	ns
Small	1.01 (0.93–1.09)	1.13 (1.02–1.19)*	1.10 (0.95–1.27)	1.15 (1.02–1.22)*	ns
**Birth interval-months**					
<18	1.00	1.00	1.00	1.00	1.00
18–24	0.92 (0.82–1.04)	0.85 (0.74–0.97)*	0.84 (0.69–1.03)	0.95 (0.80–1.12)	0.92 (0.67–1.26)
>24	0.88 (0.79–0.98)*	0.85 (0.75–0.95)*	0.87 (0.73–1.03)	0.94 (0.81–1.10)	0.87 (0.67–1.13)
**Birth order**					
1–4	1.00	1.00	ns	ns	1.00
5 +	0.44 (0.40–0.49)*	0.44 (0.39–0.48)*	ns	ns	1.31 (0.69–1.48)
Region					
Batha	1.00				
Borkou/Tibesti	1.57 (1.19–2.07)*				
Chari Baguirmi	1.49 (1.20–1.84)*				
Guera	1.27 (1.02–1.57)*				
Hadjer-Lamis	1.18 (0.94–1.48)				
Kanem	1.29 (1.02–1.64)*				
Lac	1.17 (0.93–1.46)				
Longone Occidental	1.71 (1.33–2.19)*				
Logone Oriental	1.59 (1.25–2.04)*				
Mandoul	1.34 (1.04–1.71)*				
Mayo Kebbi Est	1.33 (1.04–1.68)*				
Mayo Kebbi Ouest	1.32 (1.04–1.67)*				
Moyen Chari	1.39 (1.08–1.78)*				
Ouaddai	1.09 (0.86–1.39)				
Salamat	1.33 (1.07–1.65)*				
Tandjile	1.65 (1.29–2.11)*				
Wadi Fira	1.05 (0.82–1.36)				
N’Djamena	1.41 (1.10–1.80)*				
Barh El Gazal	1.14 (0.89–1.48)				
Ennedi	1.33 (1.02–1.72)*				
Sila	1.20 (0.94–1.53)				
Kinshasa	-	1.00			
Bandundu	-	0.96 (0.75–1.22)			
Bas-Congo	-	0.97 (0.75–1.25)			
Equateur	-	0.87 (0.69–1.11)			
Kasai-Occidental	-	0.92 (0.73–1.17)			
Kasai-Oriental	-	0.87 (0.69–1.10)			
Katanga	-	0.87 (0.69–1.09)			
Maniema	-	0.92 (0.70–1.20)			
Nord-Kivu	-	0.74 (0.56–0.96)*			
Orientale	-	0.96 (0.76–1.22)			
Sud-Kivu	-	0.89 (0.69–1.14)			
Agadez	-	-	-	1.00	
Diffa	-	-	-	1.04 (0.71–1.51)	
**Dosso**	-	-	-	1.33 (0.99–1.78)	
Maradi	-	-	-	1.19 (0.89–1.59)	
Tahoua	-	-	-	1.18 (0.88–1.58)	
Tillaberi	-	-	-	1.19 (0.89–1.60)	
Zinder	-	-	-	1.23 (0.93–1.66)	
Niamey	-	-	-	1.37 (1.01–1.89)*	

*Significant at p<0.05;

HR = hazard ratio; ns = not significant in crude mode

Women in higher economic class had significant reduction in under-five mortality when compared to poorest women, after adjusting for confounders in all study location. Marital status was significantly associated with under-five mortality in sub-Sahara Africa countries. Not currently married women had increase in under-five mortality when compared to women who are currently married or in union, after adjusting for other covariates (Chad- HR = 1.11, 95%CI = 0.97–1.25; DR Congo- HR = 1.24, 95%CI = 1.11–1.40; Mali- HR = 2.43, 95%CI = 1.63–3.64; Niger- HR = 1.59, 95%CI = 1.24–2.03; Zimbabwe- HR = 1.33, 95%CI = 1.06–1.67). For children characteristics, results on birth type showed that multiple births had higher under-five mortality, compared to singleton after controlling for covariates. Similarly, reduction in mortality was obtained from female children compared to the male counterpart. Furthermore, caesarean section mode of delivery was significantly associated with under-five mortality (Chad- HR = 1.32, 95%CI = 1.00–1.77; DR Congo- HR = 1.20, 95%CI = 1.01–1.43; Mali- HR = 1.42, 95%CI = 1.08–1.85; Niger- HR = 1.43, 95%CI = 1.06–1.92; Zimbabwe- HR = 1.49, 95%CI = 1.03–2.15). Smaller size of baby showed significant increase in under-five mortality when compared to large size, after adjusting for other covariates. Also, longer birth interval and higher birth order had significant reduction in mortality controlling for other covariates. The geographical provinces of study countries were associated with under-five mortality after controlling for other covariates.

The results spatial stratified heterogeneity q-statistic [17] is summarized in Table 3. When we applied factor detector to determine the influence of three social economic factors on under-five mortality rate in Chad, the power of determinant (q-statistic) values ranked as follows: illiteracy rate (0.172, p = 0.121) > unemployment rate (0.125, p = 0.253) > poverty rate (0.061, p = 0.608), suggesting that illiteracy rate tended to have highest influence on spatial pattern of under-five mortality rates, however, none of the reached statistically significant level. In DR Congo and Mali, poverty rate tended to have highest influence on spatial pattern followed by illiteracy and unemployment rate. In Niger, poverty rate tended to have highest influence on spatial pattern followed by unemployment and illiteracy rate. While in Zimbabwe, illiteracy rate tended to have highest influence on spatial pattern followed by poverty and unemployment rate.

**Table 3:** Geographical factor detector model.

	Poverty rate		Illiteracy rate		Unemployment rate	
	q-statistic	p-value	q-statistic	p-value	q-statistic	p-value
Chad	0.061	0.608	0.172	0.121	0.124	0.253
DR Congo	0.055	0.515	0.023	0.840	0.042	0.631
Mali	0.627	0.111	0.470	0.296	0.393	0.411
Niger	0.309	0.419	0.248	0.541	0.289	0.457
Zimbabwe	0.292	0.441	0.395	0.328	0.184	0.626

## Discussion

We investigated the trends of under-5 mortality rate by describing mortality pattern for five sub-Sahara (SSA) Africa countries. Also, we provided in-depth information on the determinants under-five mortality for each country using datasets between 2012 and 2015. The country-level variations in the under-5 mortality found in this study further re-emphasized the need that entire region can hardly be considered as a uniform entity where the same programme and policy covers all. Regardless that there has been decline in under-5 mortality levels across SSA region, the rate of decline varied substantially among the five countries under study. Though the results are presented for various years based on the data availability, the rate is still as high as 133 deaths per 1,000 live births in one of the countries under study. This is similar to the findings of previous studies [2,4,15].

We explored the possibility of regional differentials in under-5 mortality rate and its associated factors. Again, each country could have obtained different levels of children survival intervention programmes and policies because international donors and Non-Government Organizations (NGOs) work in different levels of the region. In addition, each country would have responded in different ways to the intervention programmes depending on the support of the government, programme ownership at community level to ensure sustainability, values, beliefs and norms. Previous studies have shown disparities in maternal and child health care services utilization across regions within a State [18–20]. The findings, revealed that educational level, maternal age, place of residence, economic status, and marital status amongst other children characteristics contributed to under-five mortality rate in the region. Significant reductions in under-five mortality were reported among children born to women with higher educational level, women who had spousal support, been married or currently in union, women with longer birth interval, women who had normal delivery, children with normal birth weight and women who resided in urban areas. Studies showed that women with higher education or economic are likely to be better empowered to take advantage of child survival interventions programmes. Their healthcare utilization would have remained substantially high over time and therefore appearing to be benefiting from any new intervention [8,13,18].

Similarly, some disparities across different countries could also be based on differentials in certain bio-demographic variables such as maternal age at child's birth, type and mode of birth, the interval of birth as risk factors were not all consistent across all countries. These may explain why the hazards in mortality varied in some countries. There is need to increase the uptake of health care services for children and mothers alike as reflected by the non-significance of some healthcare-related variables. In the SSA region, similar factors such as social, cultural, and economic factors could affect morbidity, mortality, fertility and children care practices [21–23]. Therefore, it can be argued that regardless of the high premium on children health care services, some of the baseline policy practices have not favored child survival in SSA countries. The gap in value for children and survival level is a major issue that should receive urgent attention from health policy makers and other stakeholders. Notably, these factors include education of women in low economic status, inadequate access to improved health care services, unsafe fertility practices [24–30].

### Strengths and limitations

In this study, we used multi-national representative large data sets which provide generalizable estimates. Another major strength of this study is that it represented a crucial effort to provide clear pattern of under-five survival across SSA region. However, it should be noted that the probability of under-five death rate was estimated based on the available data. Although effective registration system is needed to reveal the true mortality rates, but this is currently non-functional in several SSA countries. Using a cross-sectional study for data collected across various countries at different points in time may bias the comparisons of under-five mortality trend.

## Conclusion

The findings from this study have shown that despite concerted effort by government and several stakeholders in health to improve childhood survival, the rate of under-5 mortality is still high. The factors associated with childhood death as identified in this study, provide important information that can help in planning intervention programmes for under-5 survival in SSA region and other resource-constrained settings particularly in other Africa regions with common socio-economic and demographic features. From policy perspective, education and socio-economic development advocacy could be a prominent approach to tackle the burden of child mortality. Also, the position of bio-demographic factors such as birth type, birth interval, the mode of delivery, amongst others as prominent risk factors for under-five mortality should be addressed through effective design of timely and efficient intervention aimed at reducing childhood mortality in SSA countries. Future researchers should consider estimating relative risks from the latent spatial component shared by the outcome of interest and a condition-specific component.

## Abbreviations

ANC: Antenatal care
DHS: Demographic Health Survey
IRB: Institutional Review Board
MCIS: Multiple Indicator Cluster Survey
MMR: Maternal Mortality Ratio
SDGs: Sustainable Development Goals
